# Analysis of Health Behaviors and Personal Values of Childless Women, Pregnant Women and Women Who Recently Delivered

**DOI:** 10.3390/ijerph15030411

**Published:** 2018-02-27

**Authors:** Grzegorz Józef Nowicki, Patrycja Misztal-Okońska, Barbara Ślusarska, Ewa Rudnicka-Drożak, Magdalena Młynarska, Artur Czekierdowski

**Affiliations:** 1 Department of Family Medicine and Community Nursing, Medical University of Lublin, Staszica 6 Str., PL-20-081 Lublin, Poland; basiaslusarska@gmail.com; 2 Department of Emergency Medicine, Medical University of Lublin, Staszica 6 Str., PL-20-081 Lublin, Poland; patrycja.okonska@umlub.pl (P.M.-O.); magda_mlynarska@tlen.pl (M.M.); 3 Department of Family Medicine, Medical University of Lublin, Langiewicza 6A Str., PL-20-032 Lublin, Poland; edrozak@poczta.onet.pl; 4 Department of Gynecological Oncology and Gynecology, Medical University of Lublin, Staszica 16 Str., PL-20-081 Lublin, Poland; a.czekierdowski@umlub.pl

**Keywords:** pregnancy and preconception, health behaviors, preconceptional lifestyle, personal values, symbols of happiness

## Abstract

Preconception lifestyle modifications and reduction of several known risk factors may have an influence on future pregnancy outcomes. The aim of the study was to analyze health behaviors and personal values as well as to assess the relationship between these factors in women without children, in pregnant women and in women who had already delivered babies. The questionnaire survey included the Health Behavior Inventory (HBI), the Personal Value List (PVL) and sociodemographic data and was conducted in 538 women. These women were divided into three groups: women who had recently delivered (*n* = 235), pregnant women (*n* = 121) and childless women (*n* = 182). Pregnant women demonstrated a significantly higher level of declared health behaviors, and also, they rated higher on the subscales values “positive mental attitude” and “health practices”, in comparison to women who had recently delivered and to childless women. In all tested groups, the highest rated personal value was “a successful family life”, while the most appreciated symbol of happiness was “love and friendship”. Our results suggest that the system of values and the perception of happiness symbols may influence women’s health behaviors. Positioning “health” in the hierarchy of personal values as the most important one may facilitate the introduction of healthy behaviors. This, in turn, could reduce several adverse pregnancy outcomes that are potentially modifiable with changing preconception health attitudes. Our results also identify several unanswered questions and highlight areas where new research is needed.

## 1. Introduction

Existing studies suggest that health may be regarded as an instrumental value and tool that can be used to gain other values that are important to the individual. Adults who assign a high value to their health and who are convinced of their personal influence on health status usually show behavioral attitudes directed towards maintaining and improving their general health status. It is well known that women and men may have different attitudes towards health care, preventive health care, health-threatening behaviors and unhealthy behaviors [1]. Stereotypes of the female and male psychological characteristics as well as stereotypes of gender roles are reflected in patterns of behavior of both sexes, including treating health as a highly appreciated value. Women are generally characterized by having a greater interest than men in their own body and their health in particular [2,3,4]. Additionally, women usually present a better understanding of the need for prevention and treatment of diseases. In general, they employ these beliefs not only towards their own bodies but also to support their families and their social environment. The female social role of a mother and a housewife is also linked to a range of health behaviors that are more commonly practiced by women than men [5,6]. On the other hand, women increasingly demonstrate a willingness for some stereotypically male-dominated social roles. The examples are well reflected in their health behaviors, such as the use of psychoactive substances—alcohol or smoking [7,8]. Lyons et al. and Hutton et al. [8,9] found that excessive drinking in women was associated with seeking pleasure, empowerment, independence and attention from the opposite sex. Toll et al. [10] showed that smoking in women may be associated with attempts to increase femininity and sexual attraction, or even may be a sign of rebellion.

The practical dimension of health care is the realization of health-promoting behaviors in life. Several studies have confirmed that health promoting behaviors related to both health and disease prevention and treatment are more frequently found in women compared to men [11,12,13]. The behavioral health of women is at least partially determined by various demographical and cultural factors, such as socioeconomic status [14,15], age, education [16] or type of work [17,18]. Cultural conditioning, as a system of norms, beliefs and patterns of conduct, also plays an important role in the determination of personal health behaviors [19,20]. It has been suggested that systems of preferred values and norms not only affect health behaviors, but also may change the frequency of intentional childlessness occurring in Poland [21]. Importantly, knowledge about the age-related decline in female fertility is still not satisfactory [22].

The influence of personal values on the health behaviors presented by an individual has not been sufficiently investigated. Value is a psychological construct that guides the selection and assessment of behaviors in daily life choices [23]. What is more, values are linked with other psychological features, such as personality, interests or attitudes [24]. Personal values are trans-situational goals or motivations that affect attitudes, and they are expressed through behaviors; they are considered more stable than attitudes, and thus, can constitute a more consistent predictor of health behaviors. Schwartz [25] delineates values as “concepts or beliefs, pertaining to desirable end states or behaviors, transcendent of specific situations, guiding selection or evaluation of behaviors and events, and… ordered by relative importance”. Previous research has shown that, men and women differ in terms of their hierarchies of values [26,27] and the influence of their values on health-related behaviors, e.g., in the range of dietary choices [28], alcohol consumption and behaviors related to maintaining normal body mass [29]. According to the Public Opinion Research Centre (CBOS), [30] who conducted among a representative sample of Polish citizens, the values of “family happiness” and “health” are the most significant ones for the Polish population. Subsequent places were taken—according to the order indicated—by integrity, professional work, peace, respect for religious beliefs of others, friendship and education. Relatively lesser appreciation in the hierarchy of values that are particularly cherished in life has been assigned to prosperity and wealth, prosperity of homeland, a life full of adventure and excitement, freedom to proclaim one’s own views, contact with culture, success and fame and the opportunity to participate in the democratic socio-political life. Interestingly, as mentioned above, family happiness is ranked first in the hierarchy of Poles’ values, although it is more frequently emphasized by women. However, the conviction about the family as an indispensable requirement for happiness, is nowadays expressed less frequently when compared to identical studies conducted five years earlier [31]. In 2013, compared to 2008, half as many people linked happiness with having children. The authors of the study attempted to find in Polish and non-Polish research in regard to investigations on personal values of women at various stages of procreation, but we did not come across any such studies.

Female health behaviors may depend on the status of their fertility and their personal values preferences [18]. Because at least some preconceptional risk factors of adverse pregnancy outcomes could be modified, the relationship between a woman’s lifestyle and various behaviors in women who have recently delivered and childless women should be an important area of research and the subject of psychosocial monitoring. Therefore, discovering differences in approaches to health behaviors related to systems of personal values and the fundamental aspects of maternity or its deficit is a new challenge for prenatal care in women who wish to maintain their procreation health. Unravelling the new factors potentially affecting the health behaviors of pregnant women and women who have delivered babies is now a diagnostic challenge [32,33,34,35,36]. Several published studies have proven the effectiveness of various educational activities that are crucial for the planning of promotional activities [37,38]. However, there is only scant knowledge about involuntary childlessness and the seeking of possible fertility behavior modifications. The purpose of our study was to analyze health behaviors and personal values, as well as to evaluate the possible association of both of these factors in childless women, in pregnant women and in women who had already delivered and had their own babies.

## 2. Materials and Methods

### 2.1. Study Design

The study was conducted between September 2013 and May 2014 among 550 women, through a two-way paper and pencil interview (PAPI) and computer-assisted web interviewing (CAWI). The PAPI method was carried out among the female inhabitants of the Lublin voivodeship who attended the Specialized Medical Centre “INTERMED”, the Provincial Occupational Medicine Centre, the Prophylactic and Curative Centre, the “GRAVIMED” School of Birth and the “Active Mother” Maternity School. All of these units are located in Lublin, Poland. Respondents were recruited every second week of the month, following attendance at each of the aforementioned medical centers. All women expressed their written informed consent for participation in this study. Surveys through the CAWI method were conducted among women across Poland, using eight “www” web fanpages addressed to women via the Facebook platform. Links redirecting to the surveys were located on the portal “www.moje-ankiety.pl” (my-questionnaires.com) and appeared on the fanpages on the first day of the month, for a period of 9 months when the research was conducted. As many as 63.09% (*n* = 347) of female respondents participating in the research, filled out the survey via the CAWI method. The percentage female sample from individual voivodeships in Poland was representative in regard to the percentage of women in the population in particular voivodeships in Poland according to the census of the population as of 30 June 2012 [39], excluding the Lubelskie voivodeship.

Criteria for inclusion in the study group were as follows: female sex, residence in Poland, willingness, fertility and signed informed consent to participate in the study. In addition, an inclusion criterion for the group of mothers (group A) was to have at least one healthy child (without genetic defects and chronic diseases). When recruiting pregnant women (group B), only first-time mothers were considered. A criterion for inclusion in the group of women without children (group C) was not planning an offspring at the time of the research. The exclusion criterion for group A was a chronically ill, defective or disabled child in a family; for group B, previous miscarriages and/or a stillborn baby; for group C, infertility treatment. Women who completed the questionnaire surveys incorrectly or incompletely were excluded from the survey.

### 2.2. Participants

**Figure 1:**
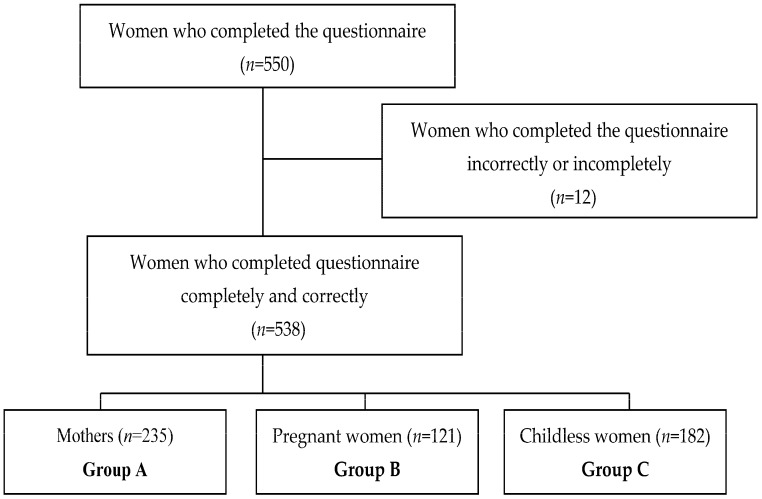
A flow-chart demonstrating the selection of studied groups.

In total, 550 questionnaires were collected, of which 538 (97.82%) were fully completed and only these were considered for further analysis. Women were divided into three groups: group A—women who recently delivered (*n* = 235), group B—pregnant women (*n* = 121) and group C—childless women (*n* = 182). The data collection flowchart is presented in Figure 1.

### 2.3. Ethics Approval

The Bioethics Committee of the Medical University of Lublin approved this study in accordance with the requirements of the Helsinki Declaration (decision number KE-0254/123/2013).

### 2.4. Questionnaires

The survey consisted of two standardized questionnaires (purchased from the Psychological Test Laboratory of the Polish Psychological Association) and a tool that assessed the socio-demographic status of the respondents:

1. Health Behavior Inventory (HBI) is a self-assessment tool that consists of 24 statements defining different health behaviors. In this inventory, an appropriate numeric value was assigned for each test item depending on how the data applied to a given respondent. These values were coded as 1, “almost never”; 2, “rarely”; 3, “from time to time”; 4, “often”; and 5, “almost always”. HBI also allows evaluation of subscales of health behaviors in four categories called “healthy dietary habits”; it also includes data on the type of food consumed. The evaluated factors included frequency of consumption of whole meal bread, fruit and vegetables, salt, and avoidance of eating food containing preservatives, “prophylactics”, or behaviors related to disease prevention, i.e., compliance with health recommendations, regular medical examinations and medical information. Next, two evaluated subscales included “health practices”, meaning everyday behaviors associated with the appropriate amount of sleep, exercise, monitoring of body weight or past times, and a “positive mental attitude”, which meant “avoidance of excessively strong emotions”, “stress” or “situations that can cause depression”. The overall result of the questionnaire, called “the index of health behaviors”, consists of a value ranging from 24 to 120 points. In general, the higher the score on this index, the higher the number of positive health behaviors. The values on this index are further subdivided into “low”, “medium” or “high” scores [40].
2. Personal Value List (PVL) was used to estimate the value that is attributed to “health” in relation to other values and personal interests important to an individual. This research tool consists of two parts: the first part includes nine symbols of happiness, defining different forms of human values, whereas the other lists 10 personal values which are rated. Respondents had to select only five out of nine symbols that were most important for them and were asked to assign the values a score from five points (the most important) to one point (the least important) for each chosen symbol. Following this selection, participants were asked to assign scores to 10 personal values, according to the above described rules. Symbols of happiness and personal values, which were not selected, were given a value of “0”. Ranks assigned by the respondent to “health” reflected the value given in this category in comparison with other values and personal properties [40].
3. Questionnaire collecting data about the respondents: a separate sheet of paper was prepared for responders’ socio-demographic data, such as age, place of residence, education, occupational status, marital status and material situation. Additionally, women who had recently delivered were asked to provide the number of children they had, whereas, mothers and pregnant women provided their preferred number of children, and childless women provided indicate the reasons behind not wanting children.

### 2.5. Statistical Analysis

The statistical analysis included the calculation of average values, standard deviations, minimums, maximums and medians for measurable values. For non-measurable parameters, the estimated frequencies and percentages were used. For measurable characteristics, the normality of the distribution was checked with the Shapiro–Wilk test. The ANOVA test was used to compare the means of HBI between groups and the Kruskal–Wallis test was used to compare the distribution of PVL items between the groups. In order to investigate the relationship between the level of health behaviors and symbols of happiness and personal values, a linear regression was used. In addition, multivariate models were performed to obtain results after controlling for covariates (age, place of residence, education level and employment status). The results of the linear regression were presented as beta coefficients with standard errors (SE) and corresponding *p*-values. A *p*-value < 0.05 was considered statistically significant. All analyses were performed with the use of Statistica v.9.1 (StatSoft, Krakow, Poland) software.

## 3. Results

### 3.1. Participants’ Characteristics

Table 1 presents the selected socio-demographic structure of respondents in terms of age, place of residence, education, occupational status, marital status, material situation, number of children in group A, preferred number of children in groups A and B, and the reasons why women from group C did not have children. The mean ages (±SD) of the studied women were 28.9 ± 4.3 years in group A, 28.7 ± 3.7 years in group B and 28.3 ± 4.5 years in group C.

### 3.2. Health Behavior of Women (HBI)

The highest mean HBI score of 86.13 (SD = 10.30) was found in women from group B. Group C had a mean HBI score of 82.44 (SD = 11.80) and group A had the lowest HBI score of 81.93 (SD = 14.51). The differences between the group of pregnant women who had higher score on the HBI scale and the groups of women who recently delivered and childless women were statistically significant (*p* < 0.05).

The highest mean score for the HBI subscale, “positive mental attitude”, was found in women from group B (3.62, SD = 0.52), followed by women from group A (3.57, SD = 0.75), and the lowest mean score in this HBI subcategory was found in women from group C (3.38, SD = 0.69). Pregnant women had significantly higher scores on the subscale, “positive mental attitude”, compared with women who had their own babies and with a group of childless women (*p* < 0.01).

“Health practices” was another HBI subscale that was different between studied groups. The highest mean level for this feature was 3.68 (SD = 0.56) found in group B, followed by the 3.40 (SD = 0.57) in group C, and the lowest mean of 3.22 (SD = 0.68) was found in respondents from group A. The use of this subscale revealed significant differences between the studied groups (*p* < 0.001). Detailed data is shown in Table 2.

### 3.3. Symbols of Happiness and Personal Values in the Studied Groups (PVL)

The results of the PVL survey in the category involving nine symbols of happiness indicated that the top priorities of studied women were “successful family life”, “good health”, “working in a dream job/profession” and “good substantive conditions”. “Successful family life” (*p* < 0.001) and “good substantive conditions” (*p* < 0.001) were scored significantly higher when assessed by women who delivered babies (group A) and by pregnant women (group C), as compared to the group of childless women. “Working in a dream job, profession” feature had a significantly increased value among women in groups C and B compared to group A (*p* < 0.05). Detailed results are presented in Table 3.

When the category involving 10 personal values was considered, the highest value was assigned to “love and friendship”, followed by “good health, physical and mental efficiency”; “happiness, contentment” and “intelligence, sharp mind”. Significant differences were found between the groups of women who delivered babies (group A) and pregnant women (group B), who valued the category “love and friendship” (*p* < 0.01) higher, compared to the group of women who had delivered babies and to women without children (group C). Interestingly, women from group C valued the category “intelligence, sharp mind” (*p* < 0.001) higher than women from groups A and B. The detailed results are presented in Table 4.

### 3.4. Relationship between Personal Values (PVL) and Health Behaviours (HBI) of Examined Women

Table 5 and Table 6 present the raw and standardized estimates of relationships between happiness and personal values (PVL) and health behaviors (HBI).

In the group of women who had recently delivered, there was a significant positive relationship between the symbol of happiness “being needed by other people” and the assessment of general HBI (b = 1.93, *p* = 0.001), and in the subscales “health eating habits” (b = 0.12, *p* = 0.001), “prophylactic behaviors” (b = 0.11, *p* = 0.001) and “positive mental attitude” (b = 0.07, *p* < 0.05). Similarly, among those women, the symbol of happiness, “good health”, had a significant positive relationship with the general HBI score (b = 2.05, *p* = 0.01) and with the subscales “health eating habits” (b = 0.12, *p* = 0.01) and “prophylactic behaviors” (b = 0.13, *p* = 0.01). Women who had recently delivered gave higher ratings to the symbols of happiness “being needed by other people” and “good health” and acquired a greater general score for both HBI and the subscales mentioned before. In the group of pregnant women, a negative relationship was observed between the general HBI score (b = −1.68, *p* = 0.01), and the subscales “health eating habits” (b = −0.11, *p* = 0.01), “positive mental attitude” (b = −0.07, *p* < 0.05) and “health practices” (b = −0.08, *p* < 0.05) and the symbol of happiness “major circle of friends”. Pregnant women for whom this symbol of happiness had a higher value received a lower rating of general HBI, as well as lower ratings for the above-mentioned subscales. In the group of childless women, higher value of the symbol of happiness “successful family life” was associated with a higher rating in the health behavior subscale “positive mental attitude” (b = 0.09, *p* = 0.01). The symbols of happiness “good substantive condition”, “adventurous life” and “fame, popularity” were not associated with any of subscales of HBI.

There was a significant positive correlation concerning the personal value “good health, physical and mental efficiency” with the general HBI score (b = 2.38, *p* < 0.001) and the subscales of HBI “health eating habits” (b = 0.12, *p* < 0.001), “prophylactic behaviors” (b = 0.18, *p* < 0.001) and “positive mental attitude” (b = 0.07, *p* < 0.01) in the group of women who had recently delivered; assigning a higher rank to this value was associated with a higher rating in the HBI score and the above-mentioned subscales of health behaviors. Moreover, among women who had recently delivered, a higher rank for “happiness, contentment” was associated with a greater general HBI score (b = 1.26, *p* < 0.05). Similarly, for women without children, the value “happiness, contentment” positively influenced the of the general HBI score (b = 1.37, *p* < 0.5) and the score for the subscale “positive mental attitude” (b = 0.09, *p* < 0.05); however, after considering the disturbing variables, this relationship did not maintain statistical significance. In the group of childless women, a significant negative relationship was observed between the rank for the personal value “sense of humor, wit” and the general HBI score (b = −2.15, *p* < 0.001) as well as the scores for the subscales “health eating habits” (b = −0.13, *p* < 0.001) and “prophylactic behaviors” (b = −0.11, *p* < 0.01). An increase in the rating of the personal value “sense of humor, wit” among childless women decreased the general HBI score and the scores for the above-mentioned subscales. In the subscale of the HBI “positive mental attitude”, a significant negative relationship with the value “sense of humor, wit” occurred only after taking into account the influence of disturbing variables (b = −0.08, *p* < 0.05). In the case of the personal value “fortune, wealth”, there was a significant negative relationship with the general HBI score in the group of women who had recently delivered and in pregnant women (b = −1.53, *p* < 0.05 vs. b = −2.49, *p* < 0.01). In addition, this value negatively influenced health behaviors in the subscale “health eating habits” (b = −0.10, *p* < 0.5) and, after considering disruptive variables, in the subscale “positive mental attitude” (b = −0.08, *p* < 0.5) in the group of women who had recently delivered. Among pregnant women, a higher rank for the value “fortune, wealth” was negatively associated with values in the subscales “prophylactic behaviors” (b = −0.11, *p* < 0.05) and “health practices” (b = −0.15, *p* < 0.01). In Group C—childless women—there was a negative correlation between personal values “fortune, wealth” and the subscale “health eating habits” (b = −0.11, *p* < 0.05).

## 4. Discussion

In recent years, research into preconceptional women’s health behaviorshas become an important area of perinatal studies. Several countries already support the use of professional psychosocial services in infertility treatment. Developing and evaluating selected psychosocial interventions is necessary to offer adequate support for women who are planning parenthood. These facts highlight the importance of modern patient-centered healthcare. General female health status varies between women due to various factors, including age, place of living and socioeconomic conditions. Women who do not receive adequate health service support are more likely to have decreased care for their preconceptional health. Several factors that may enhance positive health behaviors or could restrict a healthy lifestyle have been recently proposed. As suggested by Zielińska and Nowicka [41], reproductive health problems in women could be, at least in part, related to their socioeconomic status and social condition. The direct or indirect causes of these problems are often related to factors involved in female gender discrimination, such as lower wages, economic dependence on men, unequal distribution of family responsibilities, including their almost exclusive childcare and care for the sick, disabled and elderly. Our results indicated that several other not yet well recognized factors may affect selected conditions of women’s health. Pregnant women who regarded “wealth and fortune” as more important than other values were significantly more likely to exhibit negative health behaviors. Moreover, childless women were significantly more likely to exhibit a positive relationship between health behavior and the choice of health categories related to “happiness, contentment”. On the other hand, women who had recently delivered and pregnant women who were questioned more frequently had positive health attitudes in terms of mental health, compared to the group of childless women.

Cultural, demographic and socioeconomic considerations are important in planning and developing strategies that could effectively address the persistent disparities in various preconceptional health indicators. Several factors are responsible for these disparities, but family role is particularly important for the creation of pro- or anti-health behaviors [42]. For instance, physically active women exhibit a moderate amount of health behaviors, as reported in studies conducted by Kaczyńska-Witkowska et al. [43]. According to Weber-Rajek et al. [44], in postmenopausal women, the highest rate of health behaviors was observed among respondents who have received hormone replacement therapy (HRT). The relationship between female gender and better health behaviors is also commonly reported [11,12,45]. Women’s health behaviors in regard to cancer prevention [46] or in women diagnosed with cancer have been studied extensively [47,48,49], but the results of these studies are difficult to compare with the results presented in this paper. We have not found other studies including comparable groups of women in whom preconceptional health behaviors were assessed with the use of HBI inventory. Our studied population was characterized by a higher rate of positive health behaviors compared to the population of 315 women over 65 years of age surveyed by Młynarska et al. [50]. Bojar et al. [51] employed the same research method and analyzed levels of healthy eating habits among 88 pregnant women hospitalized in one of Lublin’s hospitals. In our study, all groups of studied women had higher levels of eating habits compared to the population studied by Bojar et al. [51], and the highest rate was found in pregnant women. Wierzejska et al. [52] found similar results and demonstrated a significant reduction or complete elimination of alcohol drinking, smoking and drinking coffee or energy drinks among pregnant women. These observations along with our own results indicate that pregnancy may be related to better health education and positive changes in the lifestyles of pregnant women.

Preconceptional health behaviors in women may be modified during preventive health visits. Such contact with health professionals may provide an opportunity to counsel the patients who are planning pregnancy as well as to women who currently are not actively trying to get pregnant. The impact of values on the level of health behaviors has been described in the literature. For instance, Pohjanheimo et al. [53] investigated the relationship between personal values and food choices. They found a positive correlation between high perceptions of health values in the hierarchy of personal values and better—i.e., “healthier”—choices of food intake. Similar results concerning the effect of values on levels of physical activity were presented by Duncan et al. [54].

The findings of our current study indicate that the categories “happiness of family life”, followed by “good health”, were the highest rated values among the studied groups of women. Ślusarska et al. [55] used the same tool (PVL) to investigate 200 nurses’ opinions on their health-related values. This group had the highest rated symbols of happiness for “good health” and “happy family life”. In another study, conducted in 50 subjects with type 2 diabetes, Derkacz et al. [56] found that the highest rated “happiness” symbol was “successful family life”. This category was followed by “good health” as the second most frequently selected. In our study, all the surveyed women indicated “love and friendship” and “good health, physical and mental” as the highest ranked values. Similarly, Rasińska and Nowakowska [57], who studied opinions of a group of nurses older than 40 years of age, found that the most esteemed personal values in this research group were “good health, physical and mental fitness” and “love, friendship”. Rudnicka-Drożak et al. [58] analyzed a group of 170 participants who were asked to choose one of the provided values that they appreciated the most highly. These values included love, money, career, religion, family, friendship and health. The most commonly chosen value was health; followed by “family”, followed by “religion”, “love”, and “money”.

Published data indicate that most studied women greatly value, and have a positive perception of, their future parenthood [34,35,36]. Our results might also suggest that women in the reproductive age value “the family” more than “health” categories. When comparing these results with other similar studies, it could be concluded that “health”, “love” and “a happy family life” are the most appreciated social values. An attempt to rank these values starting from the most to the least important would probably be unreliable and unjustified. However, it could be presumed that the nature of the research determines the final result. In our study, the nature and the title of the questionnaire used term “motherhood”; therefore, most respondents marked “love and friendship” as the cherished values. The term “happy family life” was not used in this scale; instead, “good health, physical and mental” was offered as a choice. As the nature of health status in terms of planning maternity or its deliberate avoidance is relative in nature, there is an apparent need to strengthen health education and to promote healthy behaviors among women during their preconceptional years. This is a new domain of knowledge regarding the determinants of healthy behaviors necessary to maintain the health of women. It is still to be determined whether wider implementation of knowledge on these values and changing lifestyles during preconception could influence perinatal complications rate through appropriate prevention in the future.

### Study Limitations

The most important limitations of the study are related to the relatively low number of participants and the fact that the majority of respondents had a higher education which may have affected the types of the answers. No analysis of the women’s personal values in regard to their religious preference was attempted, and this category may have influenced both the choice of motherhood and health behaviors. These factors highlight areas where new research is needed for better understanding of the personal values concerning religious life and their possible relationship with health behaviors.

## 5. Conclusions

Pregnant women are significantly more likely to present a higher overall rate of health behaviors, as assessed by the HBI test, compared to childless women and women who have recently delivered. Based on the PLV test results, the perceived value system of childless women differs from the values of pregnant women and women who have recently delivered, with a “successful family life” being rated higher than “health” in women who had recently delivered and in pregnant women. The highly ranked symbol “happy family life” from the PVL test and reported health behaviors in the HBI test did not appear to be associated with each other in the studied respondents. However, regardless of age or health status of individuals, “health” and “family” were considered timeless values and the basis of all happiness in life.

The results of our research suggest that the value system and the perception of happiness symbols can affect the health behavior of women at different stages of their reproductive life. Positioning “health” in the hierarchy of symbols of happiness and personal values as the most important might facilitate our understanding and guide strategies for the introduction of healthy behaviors. Discovering new factors that potentially affect the health behaviors of pregnant women and women who have given birth to children and the deliberately childless can be used for various educational reasons that are crucial in planning promotional activities.

## Figures and Tables

**Table 1:** Selected features of sociodemographic characteristic of studied women.

Variable	Group A (*n =* 235)	Group B (*n =* 121)	Group C (*n =* 182)
Age, years:	28.9 ± 4.3	28.7 ± 3.7	28.3 ± 4.5
<26 years	69 (29.36)	34 (28.10)	62 (34.07)
27–30 years	76 (32.34)	54 (44.63)	60 (32.97)
>31 years	90 (38.30)	33 (27.27)	60 (32.97)
Place of residence:			
City > 20.000	139 (59.15)	94 (77.69)	116 (63.74)
City < 20.000	47 (20.0)	10 (8.26)	33 (18.13)
Countryside	49 (20.85)	17 (14.05)	33 (18.13)
Education:			
Primary	0 (0.0)	1 (0.83)	1 (0.55)
Vocation	16 (6.81)	1 (0.83)	1 (0.55)
High school	63 (26.81)	8 (6.61)	34 (18.68)
Partial high school education	37 (15.74)	10 (8.26)	35 (19.23)
University	119 (50.64)	101 (83.47)	111 (60.99)
Employment status:			
White-collar worker	111 (47.23)	90 (74.38)	94 (51.65)
Manual-laborer	47 (20.0)	17 (14.05)	44 (24.18)
Physical and white-collar worker	9 (3.83)	7 (5.79)	11 (6.04)
Student	19 (8.09)	3 (2.48)	22 (12.09)
Unemployed	49 (20.85)	4 (3.31)	11 (6.04)
Marital status			
Single	13 (5.53)	7 (5.79)	98 (53.85)
Married	207 (88.09)	114 (94.21)	77 (42.31)
Divorced	15 (6.38)	0 (0)	7 (3.85)
Material situation			
Very good	17 (7.23)	8 (6.61)	11 (6.04)
Good	96 (40.85)	60 (49.59)	76 (41.76)
Average	100 (42.55)	51 (42.15)	92 (50.55)
Bad	22 (9.36)	2 (1.65)	3 (1.65)
Very bad	0 (0)	0 (0)	0 (0)
Number of children			
1 child	147 (62.55)	-	-
2 children	74 (31.49)	-	-
3 children	11 (4.68)	-	-
4 children	3 (1.28)	-	-
Preferred number of children			
1 child	18 (7.66)	9 (7.44)	-
2 children	122 (51.91)	72 (59.50)	-
3 children	80 (34.04)	36 (29.75)	-
4 children	15 (6.38)	4 (3.31)	-
I do not have children at the moment because *			
No employment, low income	-	-	155 (85.16)
Little social support	-	-	74 (40.66)
Negative attitude of employers to sick leave or maternity leave due to pregnancy and postnatal leave	-	-	78 (42.86)
The challenge of raising and educating children	-	-	35 (19.23)
Fears associated with pregnancy and childbirth	-	-	29 (15.93)
Others	-	-	9 (4.95)

Data are presented as mean ±SD or *n* (%), * respondents could choose more than one answer.

**Table 2:** Health behaviors in studied groups.

Group		General Indicator of HBI	Health Eating Habits	Prophylactic Behaviors	Positive Mental Attitude	Health Practices
A	M ± SD	81.93 ± 14.51	3.450.77	3.41 ±0.78	3.57 ± 0.73	3.22 ± 0.68
	Min–Max	36.00–116.00	1.5–5.00	1.17–5.00	1.17–5.00	1.17–4.83
	Med	83.00	3.50	3.50	3.67	3.17
B	M ± SD	86.13 ± 10.30	3.54 ± 0.60	3.51 ± 0.53	3.62 ± 0.52	3.68 ± 0.56
	Min–Max	54.00–108.00	1.50–4.67	2.33–4.83	2.00–5.00	2.17–5.00
	Med	88.00	3.50	3.50	3.67	3.67
C	M ± SD	82.44 ± 11.80	3.53 ± 0.75	3.43 ± 0.67	3.38 ± 0.69	3.40 ± 0.57
	Min–Max	56.00–113.00	1.67–5.00	1.50–4.83	1.50–4.83	1.67–4.83
	Med	83.00	3.67	3.50	3.33	3.50
Test statistic		*F* = 4.64 *	*F* = 0.99	*F* = 0.87	*F* = 6.32 **	*F* = 21.89 ***
Intergroup differences		A–B, B–C	-	-	A–C, B–C	A–B, A–C, B–C

Key: M: mean, SD: standard deviation, Med: median, F: F distribution, HBI: Health Behavior Inventory. Statistical significance is indicated by * *p* ≤ 0.05, ** *p* ≤ 0.01 and *** *p* ≤ 0.001.

**Table 3:** Comparison of symbols of happiness scoring in the studied groups.

Symbols of Happiness	Group	Mean Weight	Range (Choices in %)					
			1	2	3	4	5	0
Major circle of friends	A	1.24	13.62	11.49	14.04	5.96	4.25	50.64
	B	1.16	13.22	10.74	15.70	7.44	0.83	52.07
	C	1.23	14.29	9.89	18.68	6.04	1.65	49.45
Test statistic	H = 0.185							
Successful family life	A	4.60	2.13	4.68	3.40	17.45	69.36	2.98
	B	4.57	2.48	0.0	3.31	18.18	74.38	1,65
	C	3.80	7.69	4.95	4.95	20.87	52.75	8.79
Test statistic	H = 23.987 *** (ID: A–C, B–C)							
Working in a dream job/profession	A	1.88	17.87	15.32	22.55	13.62	3.40	27.24
	B	2.15	13.22	31.40	21.49	12.40	4.96	16.53
	C	2.30	14.84	28.02	23.63	16.48	4.39	12.64
Test statistic	H = 8.396 * (ID: A–C)							
Success in education/work	A	1.13	9.79	10.21	10.21	9.36	2.98	57.45
	B	0.56	16.53	14.05	1.65	1.65	0.0	66.12
	C	1.20	20.34	8.79	7.15	5.49	7.69	50.54
Test statistic	H = 10.946 ** (ID: B–C)							
Good health	A	3.75	3.83	9.79	11.49	42.13	29.78	2.98
	B	3.60	3.31	9.92	22.31	43.80	19.01	1.65
	C	3.64	3.30	11.53	14.84	34.07	31.32	4.94
Test statistic	H = 3.474							
Being needed by other people	A	1.55	14.47	20.00	11.06	6.81	8.08	39.58
	B	1.10	13.22	9.09	10.74	7.44	3.31	56.20
	C	1.50	8.79	13.19	20.34	6.59	5.49	45.60
Test statistic	H = 7.726 * (ID: A–B)							
Good substantive condition	A	2.18	13.62	12.77	25.53	14.89	8.51	24.68
	B	1.83	25.62	18.18	19.84	13.22	1.65	21.49
	C	1.35	18.13	15.38	9.89	12.09	1.65	42.86
Test statistic	H = 27.432 *** (ID: A–C, B–C)							
Adventurous life	A	0.69	16.17	5.96	8.51	3.40	0.43	65.53
	B	0.31	9.92	2.48	2.48	0.0	1.65	83.47
	C	0.57	8.79	7.14	1.65	1.65	4.40	76.37
Test statistic	H = 14.215 *** (ID: A–B)							
Fame, popularity	A	0.22	6.81	3.40	0.85	0.43	0.85	87.66
	B	0.02	1.65	0.0	0.0	0.0	0.0	98.35
	C	0.13	1.10	1.65	2.20	0.55	0.0	94.50
Test statistic	H = 14.443 *** (ID: A–B)							

Key: H: Kruskal–Wallis test results, ID: intergroup differences. Statistical significance is represented by * *p* ≤ 0.05, ** *p* ≤ 0.01 and *** *p* ≤ 0.001.

**Table 4:** Comparison of scores for personal values in studied groups.

Personal Values	Group	Mean Weight	Range (Choices in %)					
			1	2	3	1	5	0
Love and friendship	A	4.29	4.68	2.13	3.41	19.57	66.38	3.83
	B	4.58	2.48	0.83	3.31	14.87	76.86	1.65
	C	4.03	3.85	1.10	6.04	21.98	58.24	8.79
Test statistic	H = 12.662 ** (ID: B–C)							
Good health, physical and mental efficiency	A	3.79	2.55	5.11	8.08	45.11	32.34	6.81
	B	3.97	0.0	4.96	8.26	63.64	21.49	1.65
	C	3.70	4.95	6.59	9.89	37.91	34.07	6.59
Test statistic	H = 0.228							
Sense of humor, wit	A	1.00	9.79	5.53	14.89	6.39	1.70	61.70
	B	0.55	15.71	6.61	6.61	1.65	0.0	69.42
	C	1.04	17.03	6.59	10.44	7.15	2.75	56.04
Test statistic	H = 7.801 * (ID: A–B)							
Intelligence, sharp mind	A	1.80	8.08	17.87	24.26	11.07	3.83	34.89
	B	1.40	12.40	19.01	23.14	4.96	0.0	40.49
	C	2.06	11.54	18.13	25.82	15.39	3.85	25.27
Test statistic	H = 13.351 *** (ID: B–C)							
Knowledge and wisdom	A	1.39	9.36	14.89	17.45	7.66	3.41	47.23
	B	1.00	10.75	10.75	11.57	4.13	3.30	59.50
	C	1.39	7.14	15.94	15.38	9.34	3.30	48.90
Test statistic	H = 6.255 * (ID: A–B)							
Happiness, contentment	A	1.97	7.23	19.58	26.81	8.08	7.66	30.64
	B	2.02	7.44	31.40	27.27	8.26	3.31	22.32
	C	1.74	13.74	29.67	14.84	6.59	6.04	29.12
Test statistic	H = 4.297							
Courage, firmness	A	0.69	11.49	6.38	5.11	4.26	2.55	70.21
	B	0.40	9.92	5.78	3.31	0.0	1.65	79.34
	C	0.62	7.14	3.30	2.74	4.40	4.40	78.02
Test statistic	H = 4.832							
Kindness, consideration	A	1.21	17.02	15.32	7.66	6.81	4.68	48.51
	B	0.97	18.18	9.92	13.22	1.65	2.48	54.55
	C	0.96	12.09	9.34	9.89	5.49	2.75	60.44
Test statistic	H = 4.831							
Fine appearance, presence	A	0.46	6.81	2.55	3.40	1.28	3.83	82.13
	B	0.14	7.44	0.83	1.65	0.0	0.0	90.08
	C	0.38	6.59	5.49	4.40	1.10	0.55	81.87
Test statistic	H = 5.296							
Wealth, fortune	A	0.63	11.06	5.11	5.53	4.26	1.70	72.34
	B	0.50	13.22	7.43	4.96	0.83	0.83	72.73
	C	0.45	8.24	3.84	4.95	2.20	1.10	79.67
Test statistic	H = 3.168							

Key: H: Kruskal–Wallis test results, ID: intergroup differences. Statistical significance is represented by * *p* ≤ 0.05; ** *p* ≤ 0.01 and *** *p* ≤ 0.001.

**Table 5:** Relationship between symbols of happiness and health behaviors of surveyed women.

Symbols of Happiness	Group	Health Behaviors									
		General Indicator of HBI		Health Eating Habits		Prophylactic Behaviors		Positive Mental Attitude		Health Practices	
		b(SE)	b ^A^(SE)	b(SE)	b ^A^(SE)	b(SE)	b ^A^(SE)	b(SE)	b ^A^(SE)	b(SE)	b ^A^(SE)
Major circle of friends	A	0.26(0.62)	0.27(0.63)	0.01(0.03)	−0.03(0.02)	−0.009(0.03)	−0.02(0.02)	0.02(0.03)	0.01(0.03)	0.03(0.03)	0.02(0.03)
	B	−1.68(0.64) **	−2.17(0.66) **	−0.11(0.04) **	−0.14(0.04) **	−0.02(0.03)	−0.03(0.03)	−0.07(0.03) *	−0.10(0.03) **	−0.08(0.04) *	−0.07(0.04) *
	C	−0.44(0.60)	−0.51(0.59)	−0.04(0.04)	−0.04(0.04)	−0.03(0.03)	−0.04(0.03)	0.01(0.04)	0.02(0.03)	−0.01(0.03)	−0.02(0.03)
Successful family life	A	0.07(0.27)	0.03(0.27)	0.01(0.01)	0.01(0.01)	−0.007(0.01)	−0.01(0.01)	−0.003(0.01)	−0.004(0.01)	0.01(0.01)	0.004(0.01)
	B	−0.11(0.97)	−0.15(1.04)	0.06(0.06)	0.06(0.06)	−0.004(0.05)	−0.03(0.05)	−0.006(0.05)	−0.01(0.05)	−0.07(0.05)	−0.04(0.06)
	C	0.81(0.52)	0.86(0.53)	0.03(0.03)	0.03(0.03)	0.02(0.03)	0.03(0.03)	0.09(0.03) **	0.07(0.03) **	−0.001(0.02)	0.008(0.03)
Working in a dream job/profession	A	−0.32(0.62)	−0.33(0.62)	−0.004(0.03)	−0.009(0.03)	0.004(0.03)	0.003(0.03)	−0.03(0.03)	−0.03(0.03)	−0.02(0.03)	−0.02(0.03)
	B	0.19(0.68)	0.39(0.68)	−0.01(0.04)	−0.004(0.04)	−0.007(0.03)	0.007(0.03)	−0.03(0.03)	−0.02(0.03)	0.08(0.04) *	0.08(0.04) *
	C	−0.15(0.64)	−0.73(0.64)	0.04(0.04)	0.02(0.04)	−0.005(0.04)	−0.04(0.03)	−0.03(0.04)	−0.06(0.04)	−0.03(0.03)	−0.04(0.03)
Success in education/work	A	0.15(0.61)	0.11(0.62)	−0.04(0.03)	−0.03(0.03)	0.05(0.03)	0.04(0.03)	0.02(0.03)	0.01(0.03)	0.001(0.03)	−0.004(0.03)
	B	−0.34(1.03)	−0.14(1.05)	−0.004(0.06)	0.02(0.06)	0.04(0.05)	0.07(0.05)	−0.04(0.05)	−0.03(0.05)	−0.07(0.06)	−0.08(0.06)
	C	−0.68(0.54)	−0.56(0.53)	−0.005(0.03)	−0.00001(0.03)	−0.01(0.03)	−0.007(0.03)	−0.03(0.03)	−0.02(0.03)	−0.07(0.03) *	−0.07(0.03) *
Good health	A	2.05(0.75) **	2.22(0.75) **	0.12(0.04) **	0.12(0.04) **	0.13(0.04) **	0.13(0.04) ***	0.04(0.04)	0.05(0.04)	0.06(0.04)	0.06(0.04)
	B	0.02(0.85)	−0.11(0.88)	0.038(0.05)	0.02(0.05)	−0.06(0.04)	−0.06(0.04)	0.006(0.04)	0.009(0.04)	0.02(0.05)	0.02(0.05)
	C	0.18(0.64)	0.32(0.63)	−0.05(0.04)	−0.04(0.04)	0.03(0.04)	0.04(0.03)	0.01(0.04)	0.02(0.03)	0.04(0.03)	0.04(0.03)
Being needed by other people	A	1.93(0.57) ***	1.99(0.58) ***	0.12(0.03) ***	0.13(0.03) ***	0.11(0.03) ***	0.11(0.03) ***	0.07(0.03) *	0.07(0.03) *	0.02(0.03)	0.02(0.03)
	B	0.09(0.62)	0.23(0.64)	0.006(0.037)	−0.03(0.03)	0.01(0.03)	0.02(0.03)	−0.01(0.03)	−0.007(0.03)	0.006(0.03)	−0.003(0.04)
	C	0.58(0.54)	0.75(0.53)	0.05(0.03)	0.04(0.04)	0.03(0.03)	0.04(0.03)	0.004(0.03)	0.008(0.03)	0.02(0.03)	0.02(0.03)
Good substantive condition	A	−0.96(0.57)	−0.97(0.57)	−0.03(0.03)	−0.03(0.03)	0.005(0.03)	0.002(0.03)	−0.06(0.03) *	−0.06(0.03) *	−0.07(0.03) **	−0.07(0.03) **
	B	0.63(0.67)	0.75(0.70)	0.03(0.04)	0.04(0.04)	−0.04(0.03)	−0.03(0.03)	0.05(0.03)	0.07(0.03)	0.05(0.04)	0.04(0.04)
	C	−0.39(0.59)	−0.69(0.59)	−0.05(0.04)	−0.06(0.04)	−0.02(0.03)	−0.05(0.03)	−0.05(0.03)	−0.04(0.03)	0.05(0.03)	0.04(0.03)
Adventurous life	A	1.44(0.81)	1.24(0.83)	0.08(0.04)	0.08(0.04)	0.08(0.04)	0.06(0.04)	0.04(0.04)	0.03(0.04)	0.04(0.04)	0.04(0.04)
	B	0.60(1.09)	0.71(1.11)	0.05(0.06)	0.07(0.06)	−0.08(0.06)	−0.06(0.06)	0.03(0.06)	0.03(0.06)	0.09(0.06)	0.08(0.06)
	C	1.13(0.70)	0.86(0.68)	0.07(0.04)	0.06(0.04)	0.03(0.04)	0.02(0.04)	0.05(0.04)	0.03(0.04)	0.04(0.03)	0.04(0.03)
Fame, popularity	A	−0.75(1.32)	−1.40(1.37)	−0.03(0.07)	−0.05(0.07)	0.002(0.07)	−0.02(0.07)	−0.07(0.07)	−0.12(0.07)	−0.02(0.06)	−0.04(0.06)
	B	−7.25(7.35)	−7.24(7.77)	−0.38(0.43)	−0.30(0.45)	−0.69(0.38)	−0.60(0.39)	−0.46(0.37)	−0.49(0.40)	0.33(0.04)	0.17(0.43)
	C	−0.76(1.49)	−0.78(1.46)	−0.09(0.09)	−0.10(0.09)	−0.05(0.08)	−0.05(0.08)	0.09(0.09)	0.08(0.08)	−0.07(0.07)	−0.06(0.07)

HBI: Health Behavior Inventory; b: beta coefficient; ^A^: adjusted for age, education level, place of residence and employment status; SE: standard error. Statistical significance is indicated by * *p* ≤ 0.05; ** *p* ≤ 0.01 and *** *p* ≤ 0.001.

**Table 6:** Relationships between personal values and health behaviors of surveyed women.

Personal Values	Group	Health Behaviors									
		General Indicator of HBI		Health Eating Habits		Prophylactic Behaviors		Positive Mental Attitude		Health Practices	
		b(SE)	b ^A^(SE)	b(SE)	b ^A^(SE)	b(SE)	b ^A^(SE)	b(SE)	b ^A^(SE)	b(SE)	b ^A^(SE)
Love and friendship	A	1.17(0.72)	1.25(0.73)	0.05(0.04)	0.07(0.04)	0.06(0.04)	0.06(0.04)	0.04(0.04)	0.04(0.04)	0.04(0.03)	0.03(0.03)
	B	−0.18(0.95)	−0.24(0.95)	−0.04(0.05)	−0.04(0.05)	−0.01(0.05)	−0.02(0.05)	−0.01(0.05)	−0.02(0.05)	0.03(0.05)	0.03(0.05)
	C	0.60(0.56)	0.99(0.56)	0.004(0.03)	0.01(0.04)	0.01(0.03)	0.05(0.03)	0.09(0.03) **	0.10(0.03) **	−0.01(0.03)	0.006(0.03)
Good health, physical and mental efficiency	A	2.38(0.67) ***	2.58(0.68) ***	0.12(0.03) ***	0.12(0.04) ***	0.16(0.03) ***	0.18(0.03) ***	0.07(0.03) *	0.08(0.03) *	0.04(0.03)	0.05(0.03)
	B	1.22(1.07)	1.15(1.14)	0.04(0.06)	0.001(0.07)	0.03(0.05)	0.03(0.06)	0.09(0.05)	0.10(0.06)	0.05(0.06)	0.05(0.06)
	C	0.55(0.60)	0.54(0.60)	0.006(0.04)	0.008(0.04)	0.10(0.03) **	0.10(0.03) **	−0.03(0.03)	−0.03(0.03)	0.02(0.03)	0.01(0.03)
Sense of humor, wit	A	−0.67(0.65)	−0.73(0.65)	−0.04(0.03)	−0.04(0.03)	−0.005(0.03)	−0.009(0.03)	−0.01(0.03)	−0.02(0.03)	−0.05(0.03)	−0.05(0.03)
	B	−0.80(0.95)	−0.90(0.95)	−0.07(0.05)	−0.07(0.05)	−0.08(0.05)	−0.09(0.05)	−0.05(0.05)	−0.05(0.05)	0.06(0.05)	0.07(0.05)
	C	−2.15(0.58) ***	−2.38(0.57) ***	−0.13(0.04) ***	−0.14(0.04) ***	−0.11(0.03) **	−0.12(0.03) ***	−0.07(0.03)	−0.08(0.03) *	−0.05(0.03)	−0.06(0.03)
Intelligence, sharp mind	A	−0.40(0.60)	−0.42(0.60)	−0.02(0.03)	−0.02(0.03)	−0.005(0.03)	−0.003(0.03)	−0.007(0.03)	−0.01(0.03)	−0.03(0.03)	−0.03(0.03)
	B	−0.85(0.69)	−0.65(0.72)	−0.03(0.04)	−0.02(0.04)	−0.02(0.04)	−0.01(0.04)	−0.02(0.03)	−0.01(0.04)	−0.06(0.04)	−0.06(0.04)
	C	−1.18(0.57) *	−1.20(0.56) *	−0.02(0.04)	−0.03(0.04)	−0.05(0.03)	−0.05(0.03)	−0.06(0.03)	−0.07(0.03) *	−0.06(0.03) *	−0.05(0.03)
Knowledge and wisdom	A	−1.01(0.61)	−0.97(0.62)	−0.05(0.03)	−0.05(0.03)	−0.005(0.03)	−0.005(0.03)	−0.07(0.03) *	−0.07(0.03) *	−0.04(0.03)	−0.04(0.03)
	B	0.80(0.65)	0.89(0.67)	0.05(0.04)	0.05(0.04)	0.02(0.03)	0.03(0.03)	0.007(0.03)	0.01(0.03)	0.06(0.03)	0.05(0.04)
	C	−1.04(0.55)	−1.06(0.54)	−0.02(0.03)	−0.02(0.04)	−0.02(0.03)	−0.02(0.03)	−0.06(0.03)	−0.06(0.03) *	−0.06(0.03) *	−0.07(0.03) *
Happiness, contentment	A	1.26(0.58) *	1.25(0.59) *	0.04(0.03)	0.04(0.03)	0.05(0.03)	0.04(0.03)	0.05(0.03)	0.05(0.03)	0.07(0.03) **	0.07(0.03) *
	B	0.20(0.69)	0.14(0.70)	0.01(0.04)	0.01(0.04)	−0.01(0.03)	−0.01(0.03)	−0.05(0.03)	−0.05(0.03)	0.08(0.04) *	0.08(0.04) *
	C	1.37(0.58) *	0.98(0.58)	0.03(0.04)	0.05(0.04)	0.03(0.03)	0.008(0.03)	0.09(0.03) **	0.06(0.03)	0.05(0.03)	0.04(0.03)
Courage, firmness	A	−1.22(0.73)	−1.33(0.74)	−0.08(0.04) *	−0.07(0.04)	−0.03(0.04)	−0.04(0.04)	−0.04(0.04)	−0.05(0.04)	−0.06(0.03)	−0.06(0.03)
	B	−0.52(0.10)	−0.39(1.05)	−0.02(0.06)	0.005(0.06)	−0.0009(0.05)	0.02(0.05)	−0.04(0.05)	−0.05(0.05)	−0.02(0.05)	−0.04(0.06)
	C	0.42(0.64)	−0.09(0.64)	−0.001(0.04)	−0.02(0.04)	0.02(0.04)	−0.006(0.03)	0.06(0.04)	0.03(0.04)	−0.01(0.03)	−0.02(0.03)
Kindness, consideration	A	0.34(0.63)	0.37(0.64)	0.02(0.03)	0.027(0.03)	0.04(0.03)	0.03(0.03)	−0.008(0.03)	−0.006(0.03)	0.007(0.03)	0.005(0.03)
	B	−0.40(0.72)	−0.50(0.72)	−0.007(0.04)	−0.01(0.04)	−0.03(0.04)	−0.03(0.04)	−0.04(0.04)	−0.04(0.04)	0.004(0.04)	−0.002(0.04)
	C	−0.93(0.61)	−1.04(0.60)	−0.11(0.04) **	−0.11(0.04) **	−0.05(0.03)	−0.05(0.03)	0.04(0.03)	0.03(0.03)	−0.03(0.03)	−0.03(0.03)
Fine appearance, presence	A	−0.39(0.79)	−0.44(0.80)	−0.06(0.04)	−0.06(0.04)	0.04(0.04)	0.03(0.04)	−0.006(0.04)	−0.007(0.04)	−0.03(0.04)	−0.04(0.04)
	B	−2.63(1.92)	−2.09(1.97)	−0.11(0.11)	−0.06(0.11)	−0.22(0.10) *	−0.18(0.10)	0.007(0.10)	0.03(0.10)	−0.12(0.10)	−0.14(0.11)
	C	−1.40(0.94)	−1.27(0.92)	−0.14(0.06) *	−0.14(0.06) *	−0.04(0.05)	−0.02(0.05)	−0.07(0.05)	−0.07(0.05)	0.01(0.04)	0.02(0.04)
Fortune, wealth	A	−1.53(0.76) *	−1.73(0.77) *	−0.10(0.04) *	−0.11(0.04) **	−0.02(0.04)	−0.03(0.04)	−0.07(0.04)	−0.08(0.04) *	−0.06(0.03)	−0.07(0.04)
	B	−2.49(0.93) **	−2.38(0.96) *	−0.10(0.05)	−0.09(0.06)	−0.11(0.05) *	−0.11(0.05) *	−0.04(0.05)	−0.04(0.05)	−0.15(0.05) **	−0.16(0.05) **
	C	−0.60(0.83)	−1.13(0.82)	−0.11(0.05) *	−0.13(0.05) *	0.03(0.05)	−0.002(0.05)	−0.02(0.05)	−0.04(0.05)	0.005(0.04)	−0.01(0.04)

HBI: Health Behavior Inventory; b: beta coefficient; ^A^: adjusted for age, education level, place of residence and employment status; SE: standard error. Statistical significance is indicated by * *p* ≤ 0.05; ** *p* ≤ 0.01 and *** *p* ≤ 0.001.
